# How natural killer cells avoid self-destruction when killing their targets

**DOI:** 10.1371/journal.pbio.3001339

**Published:** 2021-08-04

**Authors:** Hannah Wurzer, Liza Filali, Clément Thomas

**Affiliations:** 1 Cytoskeleton and Cancer Progression, Department of Oncology, Luxembourg Institute of Health, Luxembourg City, Luxembourg; 2 Faculty of Science, Technology and Medicine, University of Luxembourg, Esch-sur-Alzette, Luxembourg

## Abstract

How cytotoxic lymphocytes are protected against their own weapons during close combat with diseased target cells is an important and long-standing question in immunology. This Primer explores the implications of a new study that provides new insights into the mechanisms by which natural killer cells avoid self-destruction.

## Teaser

How cytotoxic lymphocytes are protected against their own weapons during close combat with diseased target cells is an important and long-standing question in immunology. A study in this issue provides new insights into the mechanisms by which natural killer (NK) cells avoid self-destruction.

**Fig 1:**
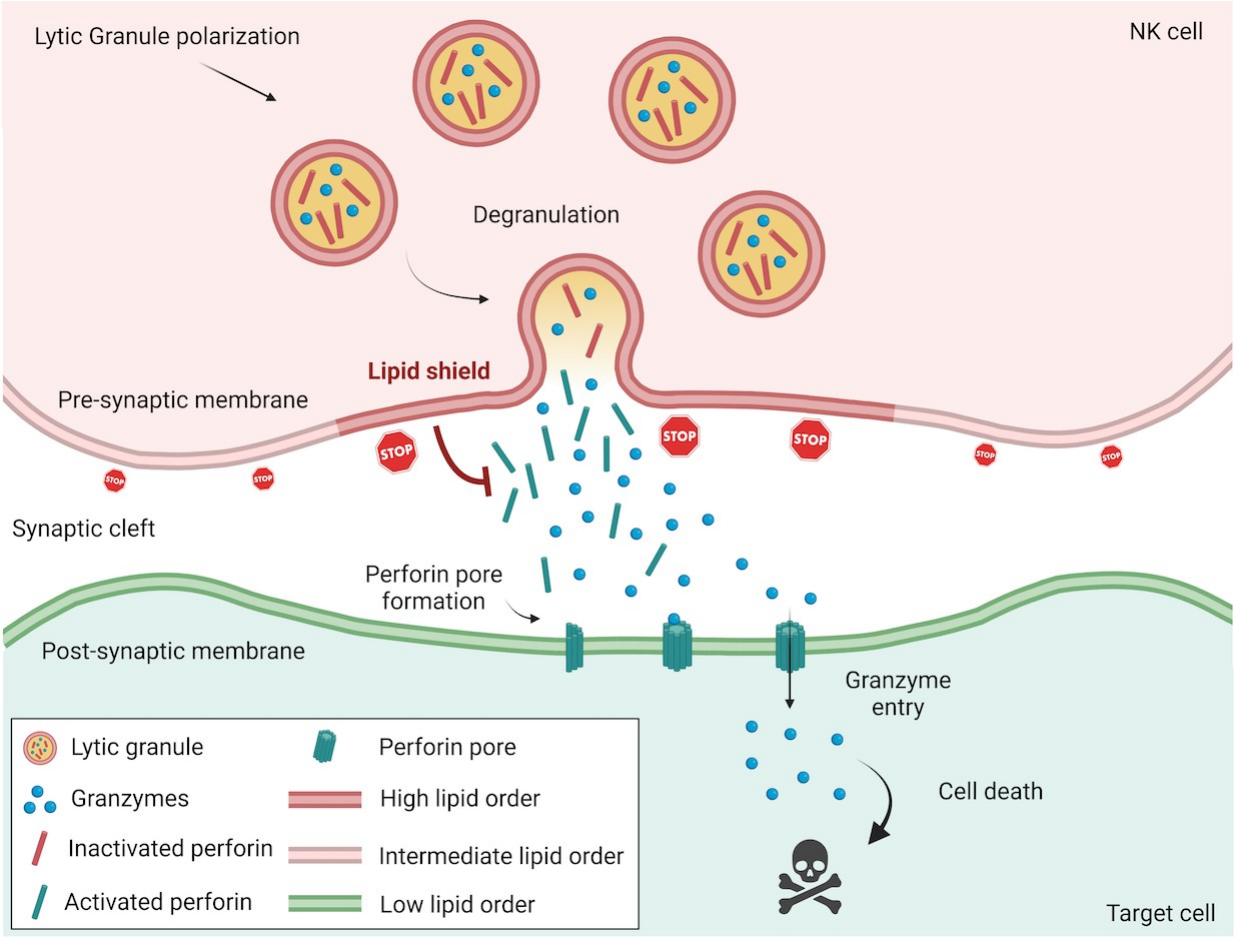
NK cells assemble a perforin-resistant lipid shield at the immunological synapse. During directional killing of virus-infected and transformed cells, NK cells establish an immunological synapse to which lytic granules are recruited before they fuse with the presynaptic membrane and release their cytotoxic content, including perforin and death-inducing granzymes, into the synaptic cleft. While perforin is inactive inside lytic granules (due to low pH and calcium concentration), it becomes fully operational in the extracellular environment where it oligomerizes in a calcium-dependent manner and creates pores into the target cell membrane. During target cell engagement, NK cells rapidly assemble a highly lipid-ordered presynaptic membrane, resulting from lipid raft aggregation that potently inhibits perforin membrane binding. Moreover, upon fusion with the NK cell plasma membrane, lytic granules, which have an intrinsically highly ordered membrane, further increase lipid packing density at the degranulation sites and thereby confer an additional layer of protection where the concentration of secreted perforin presumably peaks. NK, natural killer.

Our organism is challenged by various types of pathogens and stresses that can lead to infection and conversion of normal cells into cancer cells. The immune system is designed to recognize and eradicate aberrant cells and largely relies on killer lymphocytes, such as cytotoxic T cells (CTLs) and natural killer (NK) cells. Contrary to CTLs, NK cells do not require prior antigen exposure and clonal expansion and are pre-equipped with large amounts of cytotoxic molecules that are enclosed in secretory vesicles termed “lytic granules.” Such innate cytotoxicity (or “ready-to-kill” state) puts forward NK cells as an early line of defense against virus-infected and cancer cells and contributes to the release of antigens captured by dendritic cells for cross-presentation and priming of CTLs. Although CTLs and NK cells differ in the way they are activated and recognize diseased cells, they share a very potent killing mechanism, consisting in exocytosis of lytic granule content (a process referred to as degranulation). Among the released molecules, perforin plays a unique and critical role as it inserts pores into the target cell membrane that are indispensable for the delivery of proapoptotic granzymes [1] (Fig 1). To avoid collateral damage and destruction of surrounding healthy cells, degranulation is restricted to a highly specialized contact interface established between cytotoxic lymphocytes and their targets known as the immunological synapse. Although directional degranulation and release of cytotoxic molecules into the synaptic cleft enable selective and effective killing of target cells, they also put at risk cytotoxic lymphocytes. Synaptic perforin could act as a double-edged sword and alter the cytotoxic lymphocyte cell membrane, which is, in principle, exposed to the highest concentrations of perforin during degranulation. However, as a matter of fact, perforin-mediated pore formation predominantly occurs at the postsynaptic membrane (i.e., the target cell side of the immunological synapse), while cytotoxic lymphocyte autolysis is limited, allowing serial killing of targets [2].

In a study published in *PLOS Biology*, Li and Orange [3] explore the fascinating question of the survival advantage of NK cells over their targets. They show that, upon target cell engagement, NK cells quickly rearrange their plasma membrane lipids, resulting in a local increase of lipid packing density at the presynaptic membrane (i.e., the immune cell side of the immunological synapse) and that interfering with this process by pharmacological disruption of lipid packing is sufficient to make NK cells susceptible to their own cytotoxicity. Using a variety of complementary cell-based and synthetic cell–free assays, Li and Orange provide compelling evidence that densely packed lipid protects the presynaptic membrane from perforation by inhibiting binding of perforin. These data confirm and extend a recent study on CTLs [4] and suggest that assembly of high lipid–order presynaptic membrane is a universal mechanism by which cytotoxic lymphocytes avoid autolysis. A further novelty of Li and Orange investigations lies in the characterization of an extra layer of protection that is dependent on the degranulation process. First, they demonstrate that lytic granules are characterized by remarkably densely packed membranes with higher lipid order as compared to the NK cell presynaptic membrane. Using total internal reflection fluorescence (TIRF) microscopy, a method of choice to image dynamic processes near the cell membrane, they show that degranulation events correlate in time and space with local increase of lipid ordering, suggesting a model where cytotoxic granule fusion with the presynaptic membrane provides enhanced protection at the specific sites where perforin is secreted (Fig 1).

Tumors have evolved multiple immune evasion strategies, which are largely based on preexisting mechanisms used by healthy cells, e.g., to avoid autoimmune reactions during an acute immune response. In addition, impaired binding of perforin on the tumor cell surface has previously been reported as a potential mechanism of resistance to NK cell–mediated cytotoxicity [5]. Accordingly, Li and Orange explored lipid packing density of cancer cell membranes during NK cell attack. Very interestingly, they found that highly resistant breast cancer cells accumulate densely packed membranes at the immunological synapse, while this was not observed with more susceptible breast cancer cells. Remarkably, abrogation of postsynaptic lipid membrane packing increased the susceptibility of previously resistant breast cancer cells to NK cell–mediated killing. Therefore, Li and Orange’s study does not only expand our knowledge of immune cell biology but also contribute to a better understanding of cancer cell resistance to cytotoxic lymphocyte–mediated killing. These exciting findings lay the groundwork for future investigations and open up important questions. What is the origin of the high-order lipid membranes used to assemble the synaptic shield in cancer cells? How are these membranes recruited to synaptic region? Answering these questions could lead to the development of novel approaches to restore a potent antitumor immune response or improve the efficacy of existing immunotherapies. Recently, fast and prominent accumulation of actin filaments underneath the postsynaptic membrane has been shown to closely correlate with cancer cell resistance to NK cell–mediated lysis [6,7]. Such cytoskeletal changes likely translate into modification of membrane and vesicle trafficking, and, ultimately, result in alteration of the postsynaptic membrane composition, e.g., by promoting lipid raft aggregation and/or recruitment and fusion of vesicles. Future research should further explore lipid packing at the postsynaptic membrane in relation to actin and membrane and vesicle dynamics.

## Abbreviations

CTL: cytotoxic T cell
NK: natural killer
TIRF: total internal reflection fluorescence
